# Microbial diversity composition of apple tree roots and resistance of apple Valsa canker with different grafting rootstock types

**DOI:** 10.1186/s12866-022-02517-x

**Published:** 2022-06-03

**Authors:** Jianxun Wang, Ruolin Wang, Feng Kang, Xia Yan, Ling Sun, Nana Wang, Yufeng Gong, Xiaoning Gao, Lili Huang

**Affiliations:** 1 College of Life Science, Northwest A&F University, Yangling, 712100 China; 2 State Key Laboratory of Crop Stress Biology for Arid Areas, Northwest A&F University, Yangling, 712100 China; 3 Plant Protection and Inspection Station, Mizhi County, Shaanxi Province, Yulin, 718100 China; 4 College of Plant Protection, Northwest A&F University, Yangling, 712100 China

**Keywords:** Grafting rootstock types, Roots microbiome, *Valsa mali*, Apple Valsa canker

## Abstract

**Background:**

The composition and diversity of root microbial community are affected by plant genotypes and soil environment, which in turn affect plant growth and development. Grafting rootstock types of the apple tree can affect phenotypes in cultivation practice, but it is not clear whether grafting rootstock types can affect the composition and diversity of root microbial community and the resistance of apple tree to apple Valsa canker.

**Methods:**

To explore root microbial differences and the correlation, 16S rRNA and ITS genes were sequenced using Novaseq technology.

**Results:**

The results showed that the influence of grafting rootstock types on the composition of the root fungal community was greater than that of bacteria. And the bacterial community richness was higher in the healthy (OTUs: 1693) and dwarfing rootstock (OTUs: 1526) than in the disease (OTUs: 1181) and standard rootstock (OTUs: 1412), while the fungal community richness was the opposite. Moreover, the bacterial abundance of root zone, rhizosphere, and root endophytic microorganisms with the same grafting rootstock type exhibited a decreasing trend. Results of Nested PCR assay on soil and root tissue of *Valsa mali* showed that the content of *V. mali* in dwarfing rootstocks are lower than standard rootstocks. These results suggest that apple trees grafting with dwarfing rootstocks are more resistant to *V. mali* than standard rootstocks.

**Conclusions:**

Under different grafting types, the effect on the composition of fungal community in apple tree root was greater than that of bacteria. The bacterial community in dwarfing rootstocks is more abundant and diverse, including more beneficial microorganisms. Therefore, dwarfing rootstock is more conducive to the resistance to apple Valsa canker from biological control.

**Supplementary Information:**

The online version contains supplementary material available at 10.1186/s12866-022-02517-x.

## Introduction

Apple, one of the main economic crops in China, is planted by grafting the scions onto different rootstocks. The type of rootstock has a great influence on the quality of apple trees [1]. Using MAS marker technology, it is possible to select rootstocks genotypes such as disease resistance, dwarfing, and early maturity [2]. Studies have shown that commercial apple rootstocks have different levels of sensitivity to Apple Replant Disease (ARD), and apple rootstock G.935 might have resistance to *Pythium ultimum* [3, 4]. Studies on the microorganisms in the root zone of apple trees indicated that the diversity of the microbial community was affected by the rootstock and irrelevant with the scion [5]. Root exudates might affect the diversity of soil microorganisms [6]. Phenolics and rhizodeposits secreted by apple rootstocks can affect the composition of the microbial community in the root zone of apple trees [7].

Standard and dwarfing rootstocks are distinct patterns of grafting. The apple trees in standard rootstock orchards are tall, with strong branches and high drought resistance [8]. In dwarfing rootstock orchards, flowering and fruiting periods are relatively short, with higher planting density, higher yield, better quality fruits, and so on [9, 10]. Since the 1960s, the cultivation of dwarfing rootstocks emerged in China [11]. However, there are few studies on the utilization and cultivation techniques of dwarfing rootstock, it has not become the mainstream of apple tree grafting rootstock [12].

Apple tree also faces various abiotic and biotic stresses, including drought, low temperature, pests, microbial diseases, and so on [13–15]. In China, apple orchards are severely affected by the apple Valsa canker caused by *Valsa mali* [16, 17]. In some apple orchards, the disease rate is up to 30 percent, and even cause a large number of trees died [18, 19]. For the prevention and control of apple *Valsa* canker, chemical pesticide spraying and fruit tree peeling are generally used [17, 20]. Nevertheless, chemical control pollutes the environment, and fruit tree peeling affects the growth and development of the plant. Therefore, green biological control has gradually become a new strategy to control apple Valsa canker.

Root microorganisms form a complex network and their interactions are largely determined the beneficial traits of plants [21]. Dominant species with specific traits can perform specific functions within the microbial community [22]. For example, *Burkholderia cepacia* has a robust biological control function when acting against fungal diseases [23]. Plant growth promoting rhizobacteria (PGPR) is selected from the soil that promotes plant growth and shows a wide range of plant diseases resistance. When plants are invaded by pathogens, PGPR will produce some antibiotics, lyases, volatiles and siderophers in time to inhibit their growth, so as to reduce the damage of pathogens to plants [24–26]. Meanwhile, PGPR also help plants to tolerance abiotic stresses like salt, drought, nutrient excess or deficiency [27]. Many studies have shown that PGPR can improve the microbial community structure of plant rhizosphere soil. Wang et al. reported that microbial co-inoculants 1 (*Ensifer* sp. NYM3, *Acinetobacter* sp. P16 and *Flavobacterium* sp. KYM3) and microbial co-inoculants 2 significantly affected the indigenous soil bacterial community; notably, *Gammaproteobacteria*, *Acidobacteria*, *Nitrospirae*, and *Armatimonadetes* were significantly increased, while *Actinobacteria* and *Firmicutes* were significantly decreased by microbial co-inoculations [28]. Based on quantitative PCR and DNA sequencing network analysis, Wu et al. found that *Bacillus amyloliquefaciens* partially inhibited the nitrification process by significantly reducing ammonium-oxidizing bacteria in soil [29]. In terms of broad-spectrum biological control activity, compared with a single PGPR strain, the combination usage of PGPR strains has a better control effect on plant diseases [30, 31]. Soil microorganisms can help plants cope with the invasion of potato common scab, which is caused by *Streptomyces* spp. [32–35]. Biocontrol microorganisms can provide frontline defense against pathogen invasion [36]. Studies have found that the infection of pathogenic bacteria usually causes changes in the soil microbial community, such as that with *Oxalobacteriaceae*, *Burkholderiaceae*, and *Sphingosine bacteriaceae* in the rhizosphere, which indicates that the invading pathogens directly or indirectly influence the root bacteria [37, 38]. As we all know, diseases of woody plants are caused by many factors. And the microbial communities associated with plants are complex and dynamic, which beneficial species coexist with pathogenic species [39]. Understanding these factors and their effect on plant root microbiome will provide effective support for improving crop yield and preventing disease in the future [40]. Do root microorganisms have a certain inhibitory effect on apple Valsa canker?

Based on the above, we hypothesized that different grafting rootstocks could affect the structure of apple root microbiome, which in turn showed different resistance to apple *V. mali*. Therefore, next-generation sequencing (NGS) technologies (NovaSeq 6000) were used to study the community composition of the root microorganisms of apple trees under different grafting rootstocks and disease conditions. NovaSeq 6000 relies on Illumina’s SBS chemistry and two-color reversible terminator-based method. Combined with patterned flow cell technology [41], in excess of 3000 Gb of data can be sequenced on an S4 flow cell. The results of this study will help to further clarify the microbial community composition and diversity of different rootstocks and provide a new perspective on the control of *V. mali*.

## Results

### Sequencing data summary

The raw tags obtained by Illumina NovaSeq sequencing were spliced and underwent quality control practices to yield clean tags, and then chimera filtering was conducted to obtain effective tags for subsequent analysis. Operational taxonomic units (OTUs) were clustered using a 97% similarity cutoff with UPARSE (version 7.0.1090). A total of 2,131,276 bacterial sequences, 9,305 Operational Taxonomic Units (OTUs), and 2,728,919 fungal sequences, 3,971 OTUs were obtained in 36 DNA samples. Among bacterial, the number of OTUs that could be annotated to the database Silva132 was 8,674 (93.22%). It can be seen from the rarefaction curve of bacteria and fungi that the curves gradually became flat, indicating that the amount of sequencing data was reasonable (Fig. S1).

### Analysis of high abundance species of the apple tree root system

**Fig. 1:**
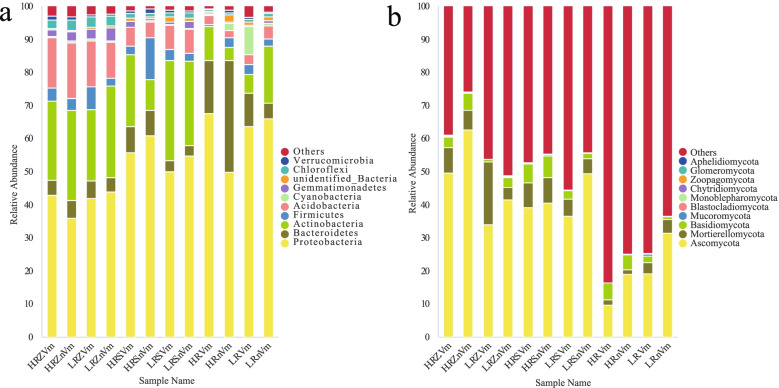
Relative abundance of the top 10 phyla of microorganisms in the all samples. The x-axis indicates communities of different samples and the y-axis represents the relative abundance within the total community. **a** Bacteria. **b** Fungi. Abbreviations: H.RZ.Vm: Disease root zone soil on standard rootstocks; H.RS.Vm: Disease rhizosphere soil on standard rootstocks; H.R.Vm: Disease root endophytes on standard rootstocks; H.RZ.nVm: Healthy root zone soil on standard rootstocks; H.RS.nVm: Healthy rhizosphere soil on standard rootstocks; H.R.nVm: Healthy root endophytes on standard rootstocks; L.RZ.Vm: Disease root zone soil on dwarfing rootstocks; L.RS.Vm: Disease rhizosphere soil on dwarfing rootstocks; L.R.Vm: Disease root endophytes on dwarfing rootstock; L.RZ.nVm: Healthy root zone soil on dwarfing rootstock; L.RS.nVm: Healthy rhizosphere soil on dwarfing rootstocks; L.R.nVm: Healthy root endophytes on dwarfing rootstocks

The root bacterial communities of apple trees in standard rootstock and dwarfing rootstock orchards were mainly *Proteobacteria*, *Bacteroidetes*, and *Actinobacteria* (Fig. 1a), and the top 10 phyla accounted for 94.73% ~ 98.68% (Table S1). Meanwhile, the phyla of fungi were relatively widespread, mainly composed of *Ascomycota*, *Mortierellomycota* and *Basidiomycota* (Fig. 1b). And especially in root endophytic fungi and its top 10 only accounted for 16.59% ~ 36.71% (Table S1).

**Fig. 2:**
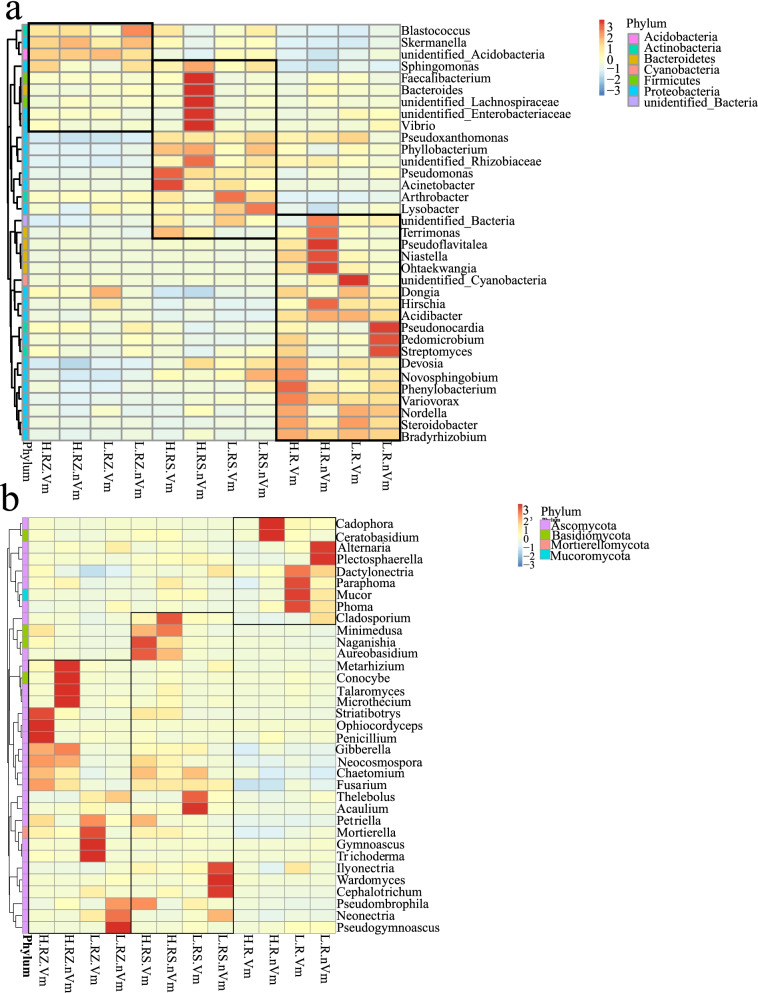
Cluster heat map of microbial species abundance at genus level. **a** Bacteria. **b** Fungi. Note: Vertical is the sample information, horizontal is the species injection path information, the clustering tree on the left is the species clustering tree. The corresponding value of the heat map is the z value obtained after standardized processing of the relative abundance of each row of species, that is, the Z value of a sample in a classification is the difference between the relative abundance of the sample in the classification and the average relative abundance of all samples in the classification divided by the standard deviation of all samples in the classification. Abbreviations: H.RZ.Vm: Disease root zone soil on standard rootstocks; H.RS.Vm: Disease rhizosphere soil on standard rootstocks; H.R.Vm: Disease root endophytes on standard rootstocks; H.RZ.nVm: Healthy root zone soil on standard rootstocks; H.RS.nVm: Healthy rhizosphere soil on standard rootstocks; H.R.nVm: Healthy root endophytes on standard rootstocks; L.RZ.Vm: Disease root zone soil on dwarfing rootstocks; L.RS.Vm: Disease rhizosphere soil on dwarfing rootstocks; L.R.Vm: Disease root endophytes on dwarfing rootstock; L.RZ.nVm: Healthy root zone soil on dwarfing rootstock; L.RS.nVm: Healthy rhizosphere soil on dwarfing rootstocks; L.R.nVm: Healthy root endophytes on dwarfing rootstocks

Due to the particularity of roots, we further analyzed niches sample communities. Through the analysis of the genus-level heat map, it can be seen that the bacterial flora of the RZ (root zone soil), RS (rhizosphere soil) and R (root endophytes) were quite different (Fig. 2a). There were certain common genera between the RZ and RS, such as, *Bacillus*, *Sphingomonas*, etc. Meanwhile, RS and R also contained some common genera, such as *Devosia*, *Novosphingobium*, *Pseudoxanthomonas*, etc. (Fig. 2a). However, the RZ and RS bacteria shared very few genera. In RZ, *Metarhizium*, *Conocybe*, and *Microthecium* were clustered in standard rootstock samples. *Neonectria*, *Pseudogymnoascus*, and *Gymnoascus* were clustered in dwarfing rootstock. In RS, standard rootstock samples mainly clustered *Cladosporium*, *Minimedusa* and Aureobasidium, dwarfing rootstock samples mainly clustered *Vishniacozyma*, *Thelebolus*, and *Acaulium*. For the root endophytic fungi, *Ceratobasidium* was the most clustered fungus in standard rootstock samples. Moreover, there were more clustered fungi in dwarfing rootstocks, including *Alternaria, Plectosphaerella, Dactylonectria, and Paraphoma* (Fig. 2b). It could be seen that the grafting rootstock types and pathogenesis had a certain influence on the composition of the fungus.

### The α diversity analysis

**Fig. 3:**
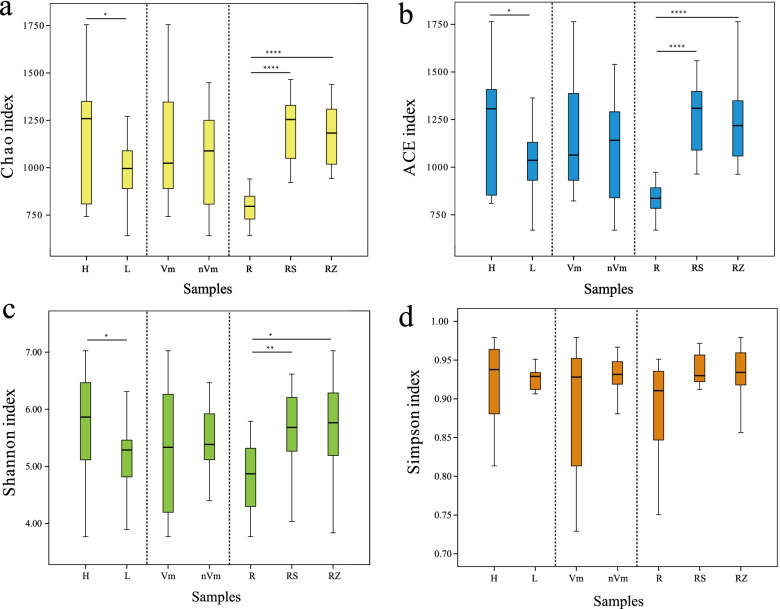
Alpha diversity analysis of the fungal communities of all samples. **a** Chao index; (**b**) Ace index; (**c**) Shannon index; (**d**) Simpson index. The x-axis indicates the sample groups and the y-axis represents the observed value of different indices based on OTU abundance. *n* = 3 for each cultivar. Bars with the different letters indicate a significant difference between means by one-way ANOVA and Duncan’s multiple test (*p* < 0.05). Values represent the mean. Error bars indicate ± standard deviation. Abbreviations: H, L represent fungal communities from “standard rootstocks”, “dwarfing rootstocks”, respectively. Vm, nVm represent fungal communities from “disease”, “health”, respectively. R, RS, RZ represent fungal communities from “root endophyte”, “root rhizosphere soil” and “root zone soil”, respectively

**Fig. 4:**
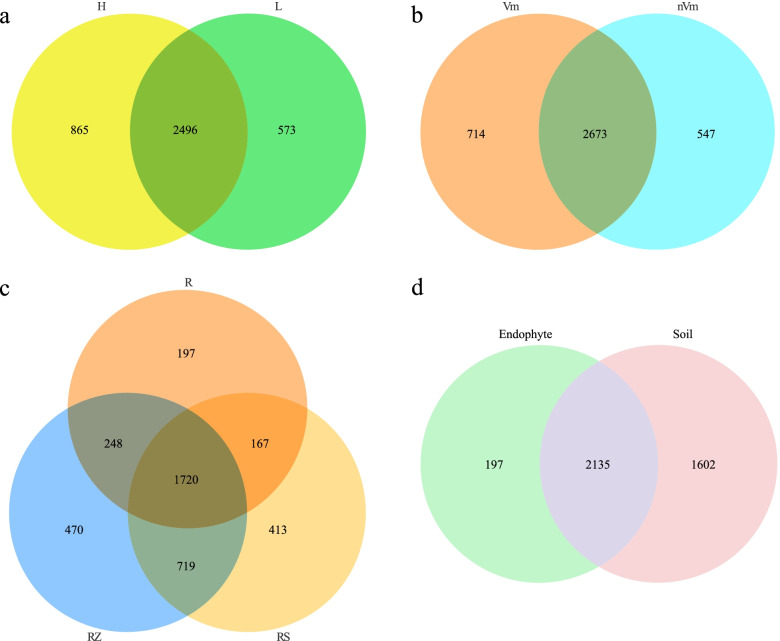
Venn diagrams illustrating the number of fungal OTUs in different groups. (**a**) Standard rootstocks (H) and dwarfing rootstocks (L); (**b**) Disease (Vm) and health (nVm); (**c**) Root endophyte (R), root rhizosphere soil (RS) and root zone soil (RZ); (**d**) Endophyte (E) and soil (root rhizosphere soil and root zone soil, S). Values represent the number of OTUs

The Chao, ACE, Shannon, and Simpson indices calculated from the fungal OTUs of all the samples indicated that α diversity of standard rootstocks was higher than that of dwarfing rootstocks (Fig. 3). Particularly, ACE and Shannon indices showed that the relative abundance and diversity of fungi in standard rootstocks were significantly higher than those in dwarfing rootstocks (Fig. 3b, 3c). Similarly, the same phenomenon was observed in bacteria, but the difference was not significant (Fig. S2). There were 2496 fungal OTUs in standard rootstock and dwarfing rootstock, and the specific OTU of standard rootstock (865) was higher than that of dwarfing rootstock (573) (Fig. 4a). However, the specific OTUs of standard rootstock (1412) was less than dwarfing rootstock (1526) in bacteria (Fig. S3a).

Based on the α diversity index, diseased and healthy had no significant effect on the richness and diversity of flora (Fig. 3, Fig. S2). The bacteria-specific OTUs of diseased samples were lower than that of healthy samples, while the OTUs of endemic fungi were more than that of the healthy group (Fig. 4b, Fig. S3b). Healthy apple trees may be enriched with certain biocontrol bacteria that increase their resistance to apple Valsa canker. Those results suggested that apple grafting rootstock types had a great impact on community diversity, followed by the whether it was infected by apple Valsa canker.

Remarkably, the richness and diversity of R were significantly lower than those in RZ and RS, as well, the richness of flora in RZ, RS, and R decreased successively (Fig. 3, Fig. S2). And there were 1720 fungal OTUs and 3992 bacterial OTUs in the three ecological niches, the specific OTUs of RZ (fungi: 470, bacteria: 1276) and RS (fungi: 413, bacteria: 858) than that in R (fungi: 197, bacteria: 538) (Fig. 4c, Fig. S3c). In addition, according to the OTUs statistics of root and soil samples, 2135 fungal OTUs and 4882 bacterial OTUs of root were found to be from soil samples, accounting for 91.55% and 90.07% of the total OTUs, respectively (Fig. 4d, Fig. S3d). This indicated that the composition of soil microorganisms has a great influence on the composition of plant root endophytic flora.

### The β diversity analysis

**Fig. 5:**
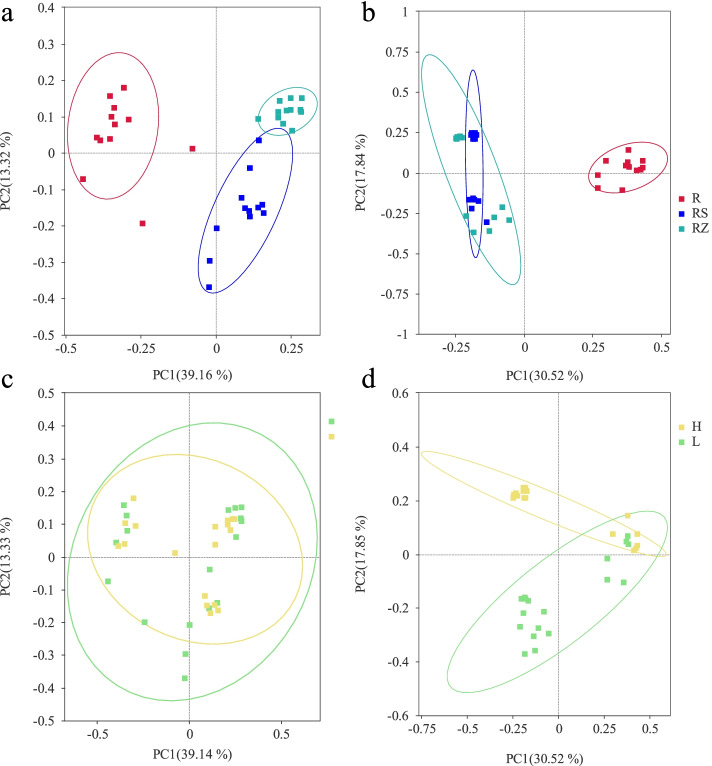
Principal Coordinate Analysis (PCoA) based on Bray–Curtis dissimilarity metrics for all samples. **a** Bacteria samples from three ecological niches (R: root endophyte; RS: root rhizosphere soil; RZ: root zone soil). **b** Fungi samples from three ecological niches (R: root endophyte; RS: root rhizosphere soil; RZ: root zone soil). **c** Bacteria samples from two planting patterns (standard rootstocks and dwarfing rootstocks). **d** Fungi samples from two planting patterns (standard rootstocks and dwarfing rootstocks). Notes: *n* = 3 for each sample. Abscissa represent one principal component, the ordinate represents another principal component, and the percentage represents the contribution of the principal component to the sample difference; each point represents a sample, and samples in the same group are represented by the same color

Principal co-ordinate analysis (PCoA) based on the Bray-Curtis distance revealed that bacteria mainly accumulated according to different niches (PC1: 39.16%, PC2: 13.32%) (Fig. 5a), and the grafting rootstock types had little effect on the differences between bacterial groups (PC1:39.14%, PC2: 13.33%) (Fig. 5c). However, the fungal samples were clustered in grafting rootstock types to a certain extent (PC1:30.52%, PC2: 17.85%) (Fig. 5d), root and two soil samples were divided into two clusters (PC1: 30.52%, PC2: 17.84%) (Fig. 5b). These results showed that the influence of grafting rootstock types on the clustering of fungi was greater than that of bacteria, while the niches had a greater effect on bacterial.

### Effects of planting patterns on fungal flora composition

**Fig. 6:**
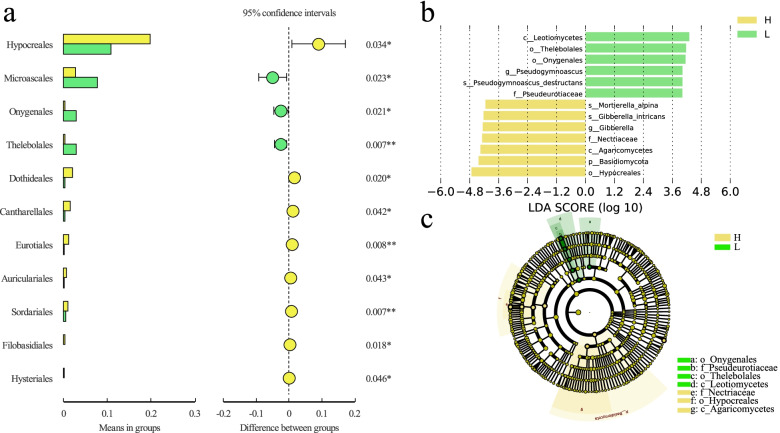
Comparison of the relative abundance of the most abundant taxa of fungi in two grafting rootstock types. **a** The 10 most abundant fungal orders in standard rootstocks (H) and dwarfing rootstocks (L) samples based on ITS amplicon sequencing. **b** Fungal LDA value distribution histogram and (**c**) evolutionary clad. Abbreviations: H, L: standard rootstocks, dwarfing rootstocks. Notes: The LDA value distribution histogram shows the species with LDA score that is greater than the set value (the default setting is 4), that is the biomarker with statistical differences between groups. The species with significant differences in abundance across different groups and the length of the histogram represents the impact of different species (i.e. LDA score). In the clade map, the circles radiating from the inside to the outside represent the classification level from the phylum to the genus (or species). Each small circle for a different classification level represents a classification at that level, and the diameter of the small circle is proportional to the relative abundance. Species with no significant differences are uniformly colored yellow, and the different species biomarker follows the group to color. The yellow node represents the microbial group that plays an important role in the yellow group, and the green node represents the microbial group that plays an important role in the green group Significantly different species of bacteria with different grafting rootstocks at species level

Since the grafting rootstock types had a significant effect on the fungal community composition, t-test was used to investigate the difference in fungal composition between the two grafting rootstock types. *Hypocreales, Dothideales, Cantharellales, Eurotiales, and Auriculariales* at the order lever were significantly more abundant in the standard rootstock than in the dwarfing rootstock orchards (*p* < 0.05) (Fig. 6a). LEfSe analysis based on LDA was performed to further determine species with significant differences in the two grafting rootstock types. In the dwarfing rootstock orchards, the relative abundance of *Ascomycota* was much higher than that of standard rootstock orchards (Fig. 6b). On the contrary, *Mucoromycota*, *Ascomycota*, and *Basidiomycota* were more prevalent in the standard rootstock orchards (Fig. 6b). Fungi with high abundance in dwarfing rootstock were closer in genetic evolution, while those with high abundance in standard rootstock were farther apart in evolution (Fig. 6c).

### Differences in composition of bacteria and fungi of soil and root endophytes.

**Fig. 7:**
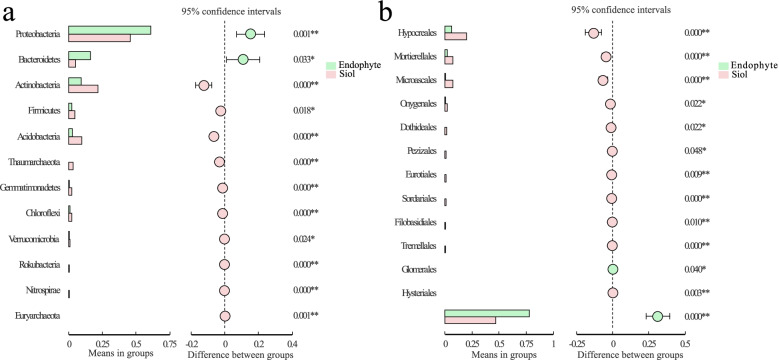
The most abundant bacterial and fungal species in root endophytes (E) and soil (root rhizosphere soil and root zone soil, S) samples based on T test. **a** Bacteria; (**b**) Fungus. ***p < 0.001, **p < 0.01, *p < 0.05. Welch’s t-test and *p*-value were corrected by the FDR method

The t-test of root endophytes and soil bacterial flora showed that *Pseudomonas frederiksbergensis, Lysobacter sp., Acidobacteria sp., * and some uncultivated bacterium in RZ and RS were significantly more abundant than those in R (*p* < 0.05) (Fig. 7a). Simultaneously, *Bradyrhizobium elkanii, Variovorax paradoxus* and *Acidobacteria* sp. were more likely to be present in roots as endophytes (*p *< 0.05) (Fig. 7a). Especially, *Bradyrhizobium elkanii* had been reported as PGPR, which could promote the growth of rice [42].

Obviously, fungi were more abundant and diverse in soil than roots (Fig. 3). For the root endophytes and soil microorganism, t-test found that *Metarhizium robertsii, Aureobasidium leucospermi, Acaulium caviariforme* and so on mainly existed in soil samples, and these were rarely present in R (Fig. 7b). Notably, the abundance of *Metarhizium Robertsii* mainly caused the differences of soil and root microbiome (Fig. S5b). Ramanpreet et.al found that *Metarhizium Robertsii* was not only rhizosphere competent but also could be associated with beneficial endophytic bacteria in roots to promote plant growth [43].

### Analysis on the difference of bacterial and fungal flora composition between disease and health

**Fig. 8:**
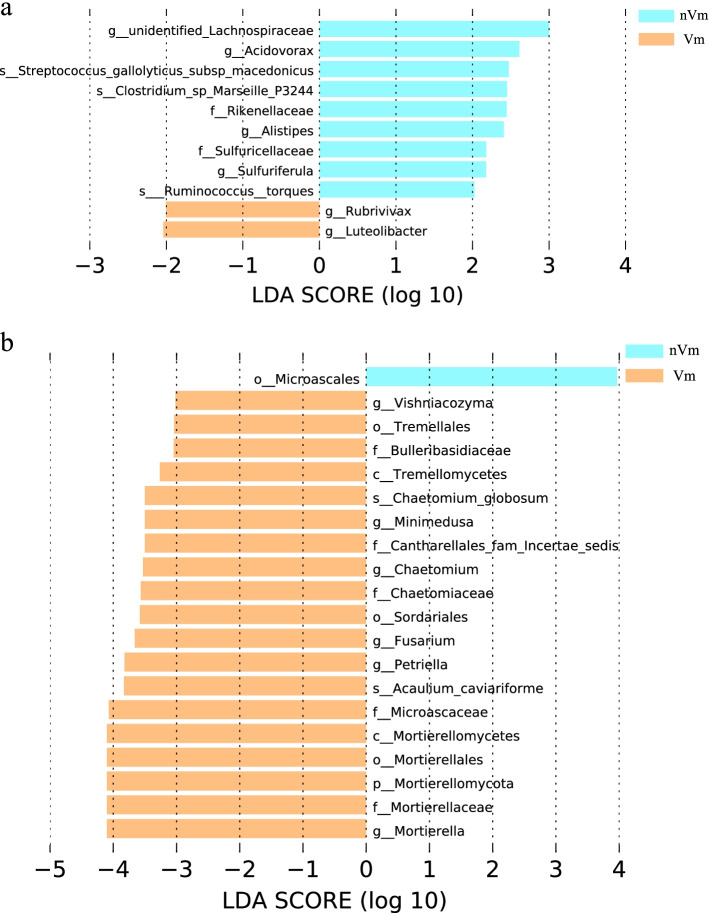
Bacterial and fungal LDA value distribution histogram in two grafting rootstock types. **a** Bacteria; **b** Fungus. Notes: The LDA value distribution histogram shows the species with LDA score that is greater than the set value (the default setting is 2 for bacteria, 4 for fungus)

LDA effect size (LEfSe) analysis was performed to further determine species with significant differences in disease and health. For bacteria, * Acidovorax and Streptococcus* were abundant in the health, while *Rubrivivax* and *Luteolibacter* were present in the disease (Fig. 8a). *Pararhizobium giardini, Streptomyces scabrisporus,* and *Vibrio ponticus* were more prevalent in the health, contributing 5.48%, 1.76% and 9.08% of the differential species, respectively (Fig. S4f). These may be part of the reason why it was not infected.

In the analysis of fungal composition difference, it was found that the fungi mainly existed in the disease (Fig. 8b). In the disease, *Vishniacozyma, Mortierellales, Minimedusa, Cantharellales and Sordariales* were more abundant, while *Microascales* were more likely to be present in the health (Fig. 8b). Among them, *Metarhizium robertsii* belonging to *Ascomycota* also played a major role in the differential flora (41.47%) (Fig. S4c).

### Function prediction of root fungi

**Fig. 9:**
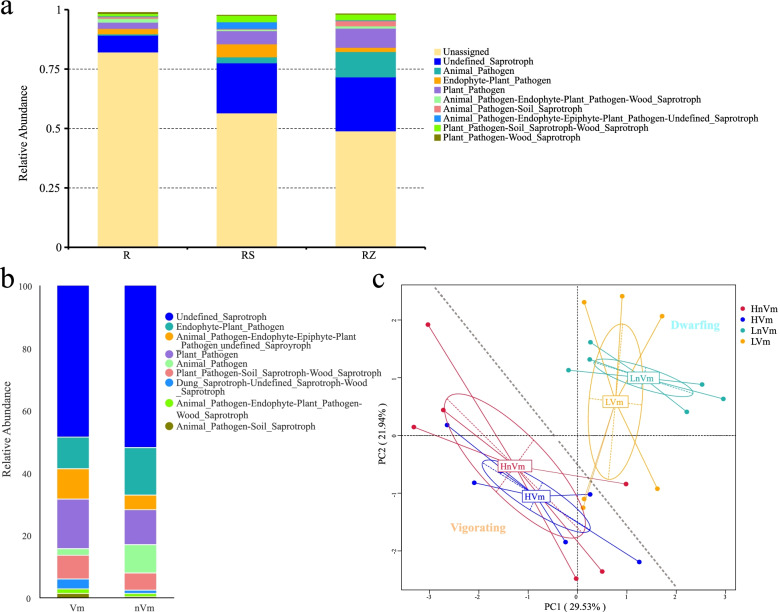
FunGuild function annotation diagram. **a** Relative abundance histogram in three niches. **b** Relative abundance histogram in disease and health; (**c**) PCA in two planting patterns with disease and health. Note: HVm: Diseased samples of vigorating rootstocks; HnVm: health samples of vigorating rootstocks; LVm: Diseased samples of dwarfing rootstocks; LnVm: Health of dwarfing rootstocks

Using FunGuild analysis, based on the OTUs of fungi, the corresponding ecological functions of the fungi could be obtained. From the perspective of the three niches, unassigned species in the R (82.21%) more than the RS (56.56%) and RZ (49.00%), while undefined-Saprotroph (R: 7.06%) was found in RS (21.08%) and RZ (22.69%) accounted for a lower proportion (Fig. 9a). This meant that there was little knowledge surrounding the function of endophytic fungi in apple trees, and the functions of many endophytic fungi were still unknown. There were many Plant_Pathogen in the soil (RS: 5.57%; RZ: 8.08%), but the proportion of endophytic fungi in the R was small (2.67%) (Fig. 9a), so it had to do with the apple tree's own immune response, and "reject" some pathogens entering the plant from the soil.

However, owing to the strong invasiveness of fungi, it could be seen that there were still some pathogenic microorganisms in endophytes. Interestingly, in apple orchards with disease, Endophyte-Plant_Pathogen (0.94%) and Plant_Pathogen (1.86%) were lower than those without disease (Endophyte-Plant_Pathogen: 3.58%; Plant_Pathogen: 3.48%) (Fig. 9b).

It could be clearly seen from the PCA that for the soil samples, similar functional fungi were based on two types of grafting rootstocks (standard and dwarfing rootstocks) for aggregation (Fig. 9c). However, in the root endophytic fungi, the functional fungi clustered together in the standard rootstock orchard, but not in the dwarfing rootstock (Fig. S5a). Endophytic fungi could be divided into two clusters according to whether the plant is healthy (Fig. S5b). These showed that the grafting rootstock types had a larger impact on the fungal function of the RS and RZ, but had a little impact on the endophytic fungus function.

### Analysis of *V. mali* in the root system

Apple Valsa canker has always been regarded as a branch disease, and there is no research on its control from the perspective of soil and root endophytes. Previous studies isolated 281 strains of fungi from Xinjiang wood, and found that they were all asexual, including *V. mali* (asexual form is *Cytospora mali*) and *Valsa sordida* (asexual form is *Cytospora chrysosperma*) [44]. The Illumina NovaSeq sequencing data from this duration showed that *Valsa mali* (*Valsa, Cytospora*) was contained in the RZ (OTUs: 27) and RS (OTUs: 20), and the content of R (OTUs: 6) was low (Table 1). In addition, the number of pathogenic fungi OTUs in the diseased (33) was indeed more than that in the health (20) (Table 1). However, its existence did not determine whether the apple Valsa canker occurs. This showed that *V. mali* exists in the soil and was "forced" to accumulate in the roots, and it would eventually become the key factor of the diseased branches of the apple tree. In this way, the combination of branch control and soil control were used to prevention the apple Valsa canker.

**Table 1:** Total OTU statistics of apple canker in all samples

Pathogen	Root	Rhizosphere soil	Root zone soil	vigorating	dwarfing	disease	health
*Valsa*	2	8	21	21	10	19	12
*Cytospora*	4	12	6	12	10	14	8
Total	6	20	27	33	20	33	20

*Note*: These numbers are the sum of the samples in that group, respectively

## Discussion

In China, standard rootstocks and dwarfing rootstocks are main grafting rootstock types. In our research, we found that there were great differences in fungi OTUs under the two grafting rootstock types. The OTUs of fungi in the standard rootstock (865) was more than the dwarfing rootstock orchards (573) (Fig. 4a). In the case of Valsa canker, the OTUs of bacteria in the health was more than the diseased, including some bacterial genera with potential biocontrol effects, for example, *Pararhizobium* (Fig. S3b, Fig. S4f). However, the fungi in the diseased orchards were more than the healthy orchards, containing genera of fungi that had the potential to cause disease, such as *Fusarium* (Fig. 4b, Fig. 8b). It could be inferred from the above phenomenon that the relationship between the incidence of apple Valsa canker and root microorganisms can be defined according to whether the beneficial microbial species are enriched. Empirical evidence and theoretical predictions suggest that species-rich communities are more resistant to pathogen invasions [45, 46]. These results also confirmed our initial hypothesis.

Differences in the composition of microbes may be related to the advantages of grafting rootstock types. Some endemic microbial species were found in dwarfing rootstock. For example, *Rhizobia* had a good inhibition on many soilborne plant pathogenic fungi belonging to different genera like *Fusarium, Rhizoctonia, Sclerotium* and *Macrophomina* [47]. *Lysobacter* can inhibit all kinds of pathogenic bacteria and fungi [48, 49]. *Variovorax paradoxus* and *Streptomyces scabrisporus* were also effective biocontrol bacteria, which could restrain the invasion of some pathogenic bacteria and have a positive effect on plant growth [50]. *Metarhizium robertsii* was not only rhizosphere competent but also displayed a beneficial endophytic association with plant roots [51]. These different microorganisms are probably a connection with the many growth advantages of dwarfing rootstock cultivation. For example, dwarfing rootstock orchards was better than standard rootstock orchards for substance absorption and transmission [52–54]. The dwarfed rootstock of the M9 variety has better specificity for the transmission of ABA, and also has a higher nitrogen absorption efficiency than other farming methods [53]. The calcium absorption rate of dwarfing rootstocks was higher than that of standard rootstocks [55, 56]. However, the phosphorus absorption efficiency of different planting methods was controversial [57, 58]. Differences in microbial community composition indicated that the dwarfing rootstock might be more resistant to *V. mali* than the standard rootstock. 

The analysis in this study found that the microflora of the three niches of the root zone, rhizosphere and root of apple trees are significant diffidence, and the abundance and richness are decrease successively. Chen et al. also showed that in mulberry trees, the richness and diversity of microbes showed a decreasing trend in root circumference, rhizosphere and roots [59, 60]. *Proteobacteria*, *Actinobacteria*, and *Acidobacteria* were the main bacterial groups in the root zone soil, while *Proteobacteria* and *Actinobacteria* in the rhizosphere soil, *Proteobacteria* and *Bacteroidetes* in the root. Meanwhile, *Ascomycota*, *Mortierellomycota* and *Basidiomycota* were the main fungal groups in the root zone, rhizosphere and root. Previous studies had shown that these microbial communities were also the dominant communities in soil and plants [61, 62]. The effect of grafting rootstock types on fungi is greater than on bacteria, while the influence of niche on bacteria and fungi is opposite. Hewavitharana et al. had shown that the type of rootstock in the greenhouse (G9.35, G.41, M.9) had a significant impact on the rhizosphere fungal community composition of apple seedlings, but had no remarkable effect on bacteria [63]. It further clarified the understanding of the relationship between grafting rootstock types and rhizosphere microorganisms. We also found *V. mali* in the soil and root endophytes, which was confirmed by sequencing data and Nested PCR. *V. mali* in the soil may from the remnants of diseased branches, or may be a pathogenic fungus existing in the soil, thereby causing apple tree disease under appropriate conditions.

Apple Valsa canker, caused by the fungus *V. mali*, is one of the most important diseases in apples [64]. Chemical pesticide spraying and fruit tree peeling were widely used to prevent and treat apple Valsa canker in China, which are environmentally unfriendly and labor-intensive [13, 14]. Through our findings, we can combine the method of biocontrol of *V. mali* with traditional control, improving the control effect of *V. mali*. Further studies will be conducted on the species with antibacterial ability in the root system of apple trees, especially the dominant species, in order to screen potential strains to control apple Valsa canker.

## Conclusions

This study mainly analyzed the relationship between the diversity of apple tree root microbial community composition and the resistance of apple Valsa canker with different grafting rootstock types. Making full use of this diversity to select superior rootstocks and effective antagonistic microorganisms are an effective method to improve agricultural value. Theoretically, it provides ideas for studying the occurrence of plant diseases, and also provides a basis for biological and ecological control of apple Valsa canker.

## Materials and methods

### Sample collection and processing

All of the samples were collected at Mizhi (110°08′75.5″ E; 37°78′42.4″ N), Shaanxi, China in November 2019. Mizhi County features a mid-temperate semi-arid climate zone. The annual average temperature is 8.1℃, the annual average rainfall is 414 mm [40].

A total of 36 samples (3 niches × 2 grafting rootstock types × 2 healthy conditions × 3 repeats, Table 2) were from "Fuji" apple trees planted for 6 years. Three niches: soil of the root zone (RZ), rhizosphere (RS), and endophyte of the root (R); two grafting rootstock types: standard rootstocks (H) and dwarfing rootstocks (L); two healthy conditions: the trees with (Vm) and without (nVm) apple Valsa canker.

**Table 2:** The sample and details

Sample	Details	Sample	Details
H.RZ.Vm	Diseased root zone soil on vigorating rootstock	L.RZ.Vm	Disease root zone soil on dwarfing rootstock
H.RS.Vm	Diseased rhizosphere soil on vigorating rootstock	L.RS.Vm	Diseased rhizosphere soil on dwarfing rootstock
H.R.Vm	Diseased root endophytes on vigorating rootstock	L.R.Vm	Diseased root endophytes on dwarfing rootstock
H.RZ.nVm	Healthy root zone soil on vigorating rootstock	L.RZ.nVm	Healthy root zone soil on dwarfing rootstock
H.RS.nVm	Healthy rhizosphere soil on vigorating rootstock	L.RS.nVm	Healthy rhizosphere soil on dwarfing rootstock
H.R.nVm	Healthy root endophytes on vigorating rootstock	L.R.nVm	Healthy root endophytes on dwarfing rootstock

*Note*: There were 12 groups of plant and soil samples, with 3 replicates per group and a total of 36 DNA samples

The planting distance of the standard rootstock orchards is 4 m × 5 m, and the dwarfing rootstock orchards were 4 m × 1.5 m. Five points around the diseased (Vm) and healthy (nVm) apple trees were selected as sampling points. First, remove the impurities and topsoil, and then collected the soil at a depth of 5–10 cm from the ground surface in a ziplock bag as the RZ. The soil and lateral roots 10–20 cm away from the ground surface were collected as the samples of RS and R, respectively. Shook the RS off the lateral roots of the apple tree, and then sieved the RS and RZ with a sterile 2 mm screen. Lateral roots were disinfected and used as the samples of apple tree endophyte, as previously described [65]. The differences caused by non-experimental variables such as edge effect, branch damage and other insect pests were excluded.

### DNA extraction and high-throughput sequencing

The genomic DNA of the samples were extracted using the TIANamp Soil DNA Kit and cetyltrimethylammonium bromide (CTAB) method, and PCR amplification was performed with 16S V3 region primers (341F: CCTAYGGGRBGCASCAG; 806R: GGACTACNNGGGTATCTAAT) and ITS region primers (ITS5-1737F: GGAAGTAAAAGTCGTAACAAGG; ITS2-2043R: GCTGCGTTCTTCATCGATGC). All PCR reactions were carried out with 15 µL of Phusion® High-Fidelity PCR Master Mix (New England Biolabs, USA), 6 µM of forward and reverse primers, and about 10 ng template DNA, the total volume is 30 µL. Thermal cycling consisted of initial denaturation at 98℃ for 1 min, followed by 30 cycles of denaturation at 98℃ for 10 s, annealing at 50℃ for 30 s, and elongation at 72℃ for 30 s. Finally, 72℃ for 5 min. Equal concentration mixing was performed according to PCR product concentration and 2% agarose gel electrophoresis was applied to purify the PCR products after full mixing. Screened the bands with sequence size of 400–450 bp and reclaimed the PCR products with the GeneJET gel recovery kit (Thermo Scientific, USA). Next, we shipped the recovered DNA to Novogene (Beijing, China) and used the TruSeq® DNA PCR-Free Sample Preparation Kit (Illumina, USA) for library construction. The library quality was preliminary determined by Qubit@ 2.0 Fluorometer (Thermo Scientific, USA), and Q-PCR (real-time PCR by using primers F: 5’- AATGATACGGCGACCACCGA-3’; R: 5’-CAAGCAGAAGACGGCATACGA-3’) was used for accurate and quantitative library detection. After the library was qualified, sequenced with NovaSeq6000. The sequence files of bacteria and fungi have been uploaded to NCBI, and the accession numbers are PRJNA675028 and PRJNA675150 respectively.

### Data processing and analysis

To facilitate analyse the differences of microbial community composition and the resistance of apple Valsa canker with different grafting rootstocks, 12 groups were established: 1) H.RZ.Vm; 2) H.RS.Vm; 3) H.R.Vm; 4) H.RZ.nVm; 5) H.RS.nVm; 6) H.R.nVm; 7) L.RZ.Vm; 8) L.RS.Vm; 9) L.R.Vm; 10) L.RZ.nVm; 11) L.RS.nVm; 12) L.R.nVm.

The Illumina Novaseq6000 is a paired-end sequencing instrument, and the reads are paired-end. We separated each sample data from the offline data according to the barcode sequences and PCR amplification primer sequences. After trimming the barcode and primer sequences, FLASH (V1.2.7, http://ccb.jhu.edu/software/FLASH/) was applied to splice, and QIIME (V1.9.1, http://qiime.org/scripts/split libraries fastq.html) was perform to filter, and FastQC (https://www.bioinformatics.babraham.ac.uk) was utilized quality control the reads of each sample. We then performed chimera filtering to obtain effective tags that could be used for subsequent analysis [66–69]. Next, Uparse (Uparse v7.0.1001, http://drive5.com/uparse) was utilized to cluster the effective tags of all samples, and the sequences were clustered into Operational Taxonomic Units (OTUs) with 97% identity by default. The most frequently occurring sequence was screened as the representative sequence of OTUs, the OTUs sequences were annotated, and the SSUrRNA database of SILVA132 (http://www.arb-silva.de) were used to annotate the bacteria species (set threshold is 0.8 ~ 1); blasted in QIIME and Unit database (v7.2, https://unite.ut.ee) were employed to perform species annotation analysis of fungi. Using MUSCLE (Version 3.8.31, http://www.drive5.com/muscle) to perform rapidly multiple sequence alignment, and obtain the phylogenetic relationship of all OTUs representative sequences. Finally, the data of each sample was normalized, and the samples with the least amount of data was used as the standard. The subsequent α diversity analysis and β diversity analysis were based on the data after the normalization. R (Version 2.15.3) was used to draw the rarefaction curve, visualize the data and draw the PCoA, analyse the species with significant differences among groups to perform the t-test among groups and plot [70, 71]. According to the results of OTUs obtained by clustering, we analyzed the common and unique OTUs among different samples (groups), and generated a Venn diagram. Then, QIIME was applied to calculate Chao, ACE, Shannon, Simpson, and Unifrac distance matrix. The LDA score of the LEfSe analysis was set to 2, 3 or 4. LEfSe is a complex algorithm, non-parametric factorial Kruskal-Wallis (KW) sum-ranktest was used to detect species with significant differences in abundance between different groups, and then Wilcoxon rank sum test was used to judge the differences between groups. Finally, linear discriminant analysis (LDA) was used to reduce and evaluate the impact of species with significant differences (LDA Score). Finally, the FUNGuild was used to predict the functions of ITS.

## Supplementary Information

**Additional file 1.**

## Data Availability

Sequence data generated and analyzed during the current study are available in the NCBI SRA, BioProject ID: PRJNA675028 and PRJNA675150, https://www.ncbi.nlm.nih.gov/bioproject/?term=PRJNA675028 and https://www.ncbi.nlm.nih.gov/bioproject/?term=PRJNA675150.

## Abbreviations

RZ: Soil of the root zone
RS: Rhizosphere
R: Endosphere of the root
Vm: Diseased apple trees
nVm: Healthy apple trees
H: Vigorous rootstocks
L: Dwarfing rootstocks
E: Root endophytes
S: Root rhizosphere soil and root zone soil
PCA: Principal Component Analysis
ITS: Internal Transcribed Spacer
MAS: Molecular Marker-Assisted Selection
ARD: Apple Replant Disease
PGPR: Plant growth promoting rhizobacteria
OTU: Operational Taxonomic Unit
PCoA: Principal co-ordinate analysis
LDA: Linear discriminant analysis
LEfSe: Linear discriminant analysis Effect Size
CTAB: Cetyltrimethylammonium bromide
