# Supplementary material for: Microbial diversity composition of apple tree roots and resistance of apple Valsa canker with different grafting rootstock types
Source: BMC Microbiol. 2022 Jun 3;22:148. doi: 10.1186/s12866-022-02517-x (PMC9164711; doi:10.1186/s12866-022-02517-x)
Supplement: Supplementary file 1 — Additional file 1. [file 12866_2022_2517_MOESM1_ESM.docx]

**Table S1**. Proportion of bacteria and fungi top 10

| **group** | **bacteria** | | **fungi** | |
| --- | --- | --- | --- | --- |
|  | **Top 10** | **others** | **Top 10** | **others** |
| H.RZ.Vm | 94.73% | 5.27% | 60.27% | 39.73% |
| H.RZ.nVm | 94.84% | 5.16% | 73.42% | 26.58% |
| L.RZ.Vm | 95.53% | 4.47% | 53.42% | 46.58% |
| L.RZ.nVm | 96.09% | 3.91% | 48.68% | 51.32% |
| H.RS.Vm | 97.11% | 2.89% | 52.52% | 47.48% |
| H.RS.nVm | 96.86% | 3.14% | 54.99% | 45.01% |
| L.RS.Vm | 97.31% | 2.69% | 44.37% | 55.63% |
| L.RS.nVm | 97.20% | 2.80% | 55.43% | 44.57% |
| H.R.Vm | 98.68% | 1.32% | 16.59% | 83.41% |
| H.R.nVm | 97.53% | 2.47% | 25.19% | 74.81% |
| L.R.Vm | 95.42% | 4.58% | 25.36% | 74.64% |
| L.R.nVm | 97.07% | 2.93% | 36.71% | 63.29% |

Abbreviations: H.RZ.Vm: RZ soil with disease on vigorating rootstocks; H.RS.Vm: RS soil with dis-eased vigorating rootstocks; H.R.Vm: endophytic bacteria with diseased vigorating root-stocks; H.RZ.nVm: RZ non-diseased vigorating rootstocks; H.RS.nVm: RS with diseased vigorating rootstocks; H.R.nVm: endophytic bacteria without diseased vigorating root-stocks; L.RZ.Vm: RZ soil with diseased dwarfing rootstocks; L.RS.Vm: diseased dwarfing rootstocks in RS soil; L.R.Vm: diseased dwarfing rootstock endophytic bacteria; L.RZ.nVm: non-diseased dwarfing rootstock RZ soil; L.RS.nVm: non-diseased dwarfing rootstocks RS soil; L.R.nVm: non-diseased endophytes on dwarfing rootstocks.


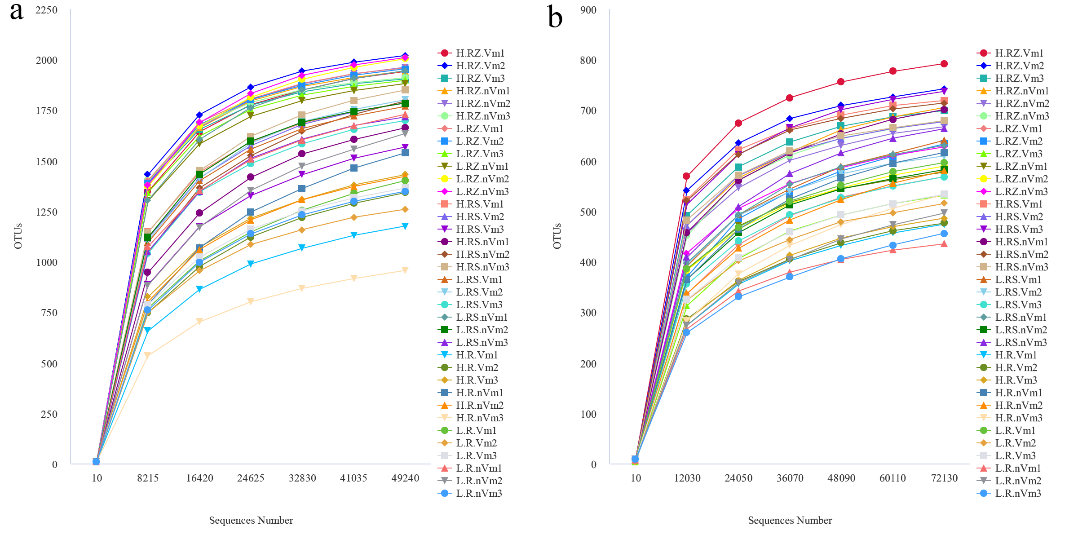


**Figure S1.** Rarefaction curves depicting the number of OTUs identified in each sample using a 97% similarity. (a) Bacterial; (b) Fungal. The x-axis indicates the number of sequences obtained from each sample. The y-axis represents the number of OTUs observed based on the Sobs index. N=3 for each sample. Different colors represent different groups.


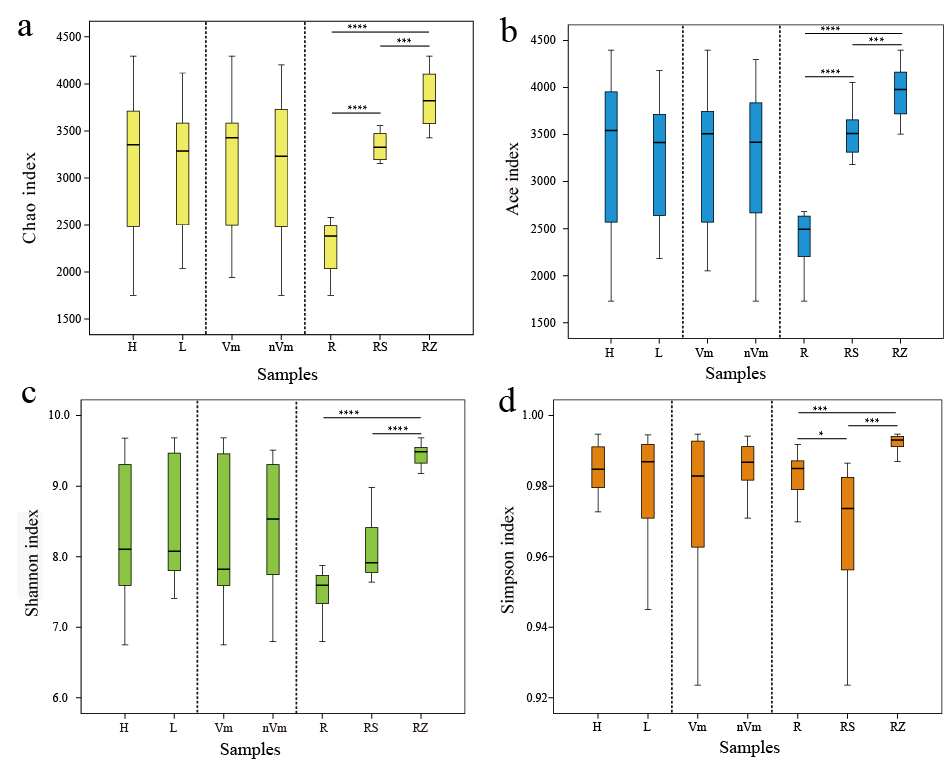


**Figure S2**. Alpha diversity analysis of the bacterial communities of all samples. (a) Chao; (b) Ace; (c) Shannon; (d) Simpson. The x-axis indicates the sample groups and the y-axis represents the observed value of different indices based on OTU abundance. n = 3 for each cultivar. Bars with the different letters indicate a significant difference between means by one-way ANOVA and Duncan’s multiple test (p < 0.05). Values represent the mean. Error bars indicate ± standard devi-ation. Abbreviations: H, L represent bacterial communities from “vigorating rootstocks”, “dwarfing rootstocks”, respectively. Vm, nVm represent bacterial communities from “disease”, “non-diseased”, respectively. R, RS, RZ represent bacterial communities from “root endophyte”, “root rhizosphere soil” and “root zone soil”, respectively.


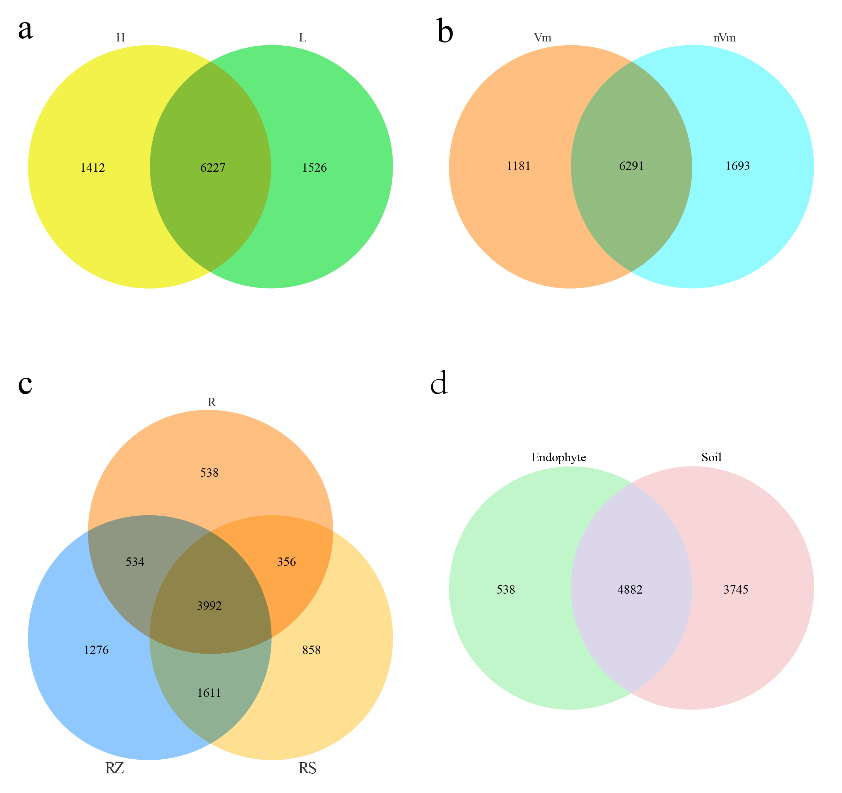


**Figure S3**. Venn diagrams illustrating the number of bacterial OTUs in different groups. (a) Vig-orating rootstocks (H) and dwarfing rootstocks (L); (b) Disease (Vm) and non-diseased (nVm); (c) Root endophyte (R), root rhizosphere soil (RS) and root zone soil (RZ); (d) Endophyte (E) and soil (root rhizosphere soil and root zone soil, S). Values represent the number of OTUs.


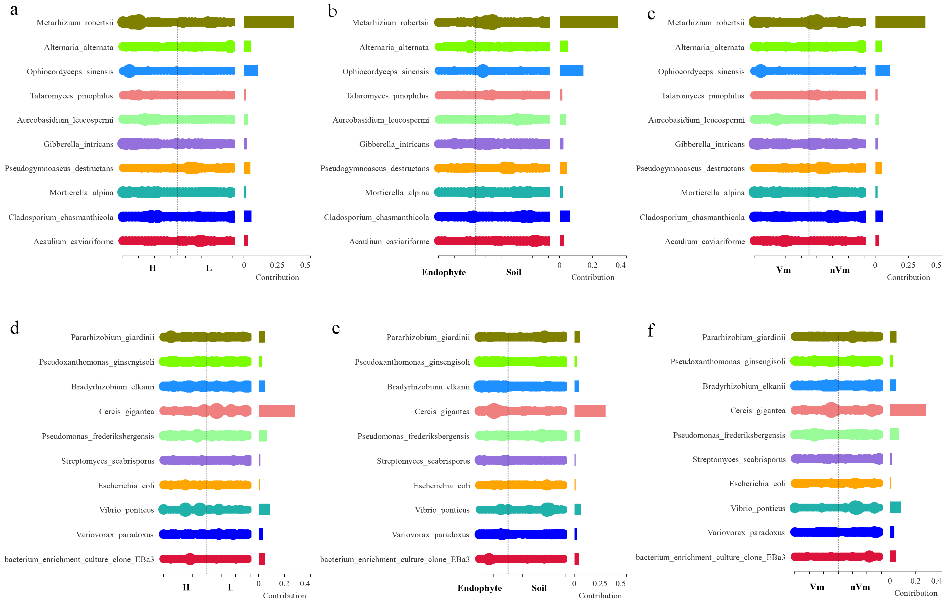


**Figure S4**. SIMPER calculation results of different groups. a, b, c. Fungai; d, e, f. Bacteria. Note: Vigorating rootstocks (H) and dwarfing rootstocks (L); B. Disease (Vm) and non-diseased (nVm); C. Root endophyte (R), root rhizosphere soil (RS) and root zone soil (RZ); D. Endophyte (E) and soil (root rhizosphere soil and root zone soil, S). SIMPER calculates the average difference be-tween all sample pairs across regions and then evaluates the relative difference in each species' contribution. Distribution patterns of rotifer communities were assessed at the intraspecific level based on bray-Curtis distance.


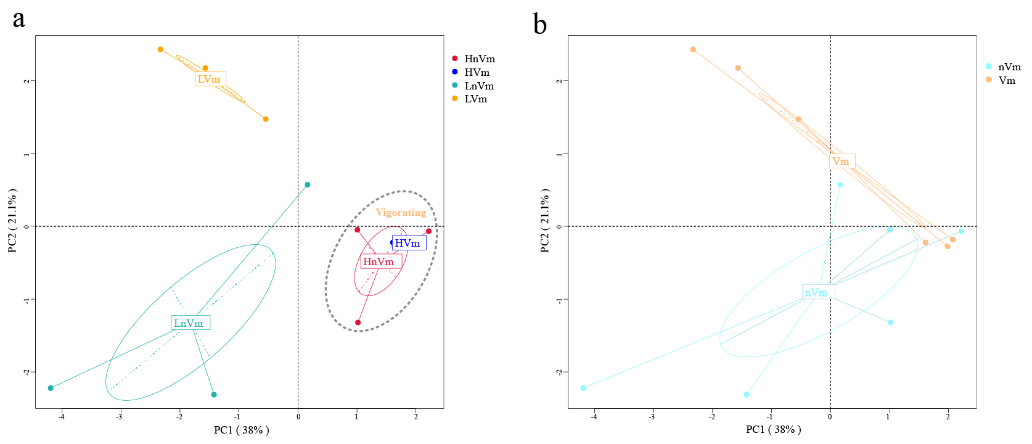


**Figure S5**. FunGuild function PCA of root endophytic fungi. (a) PCA of root endophytic fungi in two planting patterns with disease and non-diseased. (b) PCA of root endophytic fungi with dis-ease and non-diseased. Note: HVm: the samples of vigorating rootstocks with disease; HnVm: the samples of vigorating rootstocks with non-disease; LVm: the samples of dwarfing rootstocks with disease; LnVm: the samples of dwarfing rootstocks with non-disease. Vm: the samples of disease; nVm: the samples of non-diseased.
